# CheSS: Chest X-Ray Pre-trained Model via Self-supervised Contrastive Learning

**DOI:** 10.1007/s10278-023-00782-4

**Published:** 2023-01-26

**Authors:** Kyungjin Cho, Ki Duk Kim, Yujin Nam, Jiheon Jeong, Jeeyoung Kim, Changyong Choi, Soyoung Lee, Jun Soo Lee, Seoyeon Woo, Gil-Sun Hong, Joon Beom Seo, Namkug Kim

**Affiliations:** 1 Department of Biomedical Engineering, Asan Medical Center, College of Medicine, Asan Medical Institute of Convergence Science and Technology, University of Ulsan, Seoul, Republic of Korea; 2 Department of Convergence Medicine, Asan Medical Center, Asan Medical Institute of Convergence Science and Technology, University of Ulsan College of Medicine, 5F, 26, Olympic-Ro 43-Gil, Songpa-Gu, Seoul, 05505 Republic of Korea; 3 Department of Radiology, Asan Medical Center, University of Ulsan College of Medicine, Seoul, Republic of Korea; 4 Department of Radiology and Research Institute of Radiology, Asan Medical Center, University of Ulsan College of Medicine, Seoul, Republic of Korea; 5 Department of Industrial Engineering, Seoul National University, Seoul, Republic of Korea; 6 Department of Biomedical Engineering, University of Waterloo, Waterloo, ON Canada

**Keywords:** Chest X-ray, Classification, Contrastive learning, Pretrained weight, Self-supervised learning, Bone suppression

## Abstract

Training deep learning models on medical images heavily depends on experts’ expensive and laborious manual labels. In addition, these images, labels, and even models themselves are not widely publicly accessible and suffer from various kinds of bias and imbalances. In this paper, chest X-ray pre-trained model via self-supervised contrastive learning (CheSS) was proposed to learn models with various representations in chest radiographs (CXRs). Our contribution is a publicly accessible pretrained model trained with a 4.8-M CXR dataset using self-supervised learning with a contrastive learning and its validation with various kinds of downstream tasks including classification on the 6-class diseases in internal dataset, diseases classification in CheXpert, bone suppression, and nodule generation. When compared to a scratch model, on the 6-class classification test dataset, we achieved 28.5% increase in accuracy. On the CheXpert dataset, we achieved 1.3% increase in mean area under the receiver operating characteristic curve on the full dataset and 11.4% increase only using 1% data in stress test manner. On bone suppression with perceptual loss, we achieved improvement in peak signal to noise ratio from 34.99 to 37.77, structural similarity index measure from 0.976 to 0.977, and root-square-mean error from 4.410 to 3.301 when compared to ImageNet pretrained model. Finally, on nodule generation, we achieved improvement in Fréchet inception distance from 24.06 to 17.07. Our study showed the decent transferability of CheSS weights. CheSS weights can help researchers overcome data imbalance, data shortage, and inaccessibility of medical image datasets. CheSS weight is available at https://github.com/mi2rl/CheSS.

**Supplementary Information:**

The online version contains supplementary material available at 10.1007/s10278-023-00782-4.

## Introduction

Training deep learning models with medical images is very difficult. Only a few data are accessible due to a variety of problems. In producing medical data, complicated issues such as human rights of patients, copyrights of the medical doctor who processed the medical information into the usable medical data, and other legal issues are entangled. Accordingly, Health Insurance Portability and Accountability Act (HIPAA) and General Data Protection Regulation (GDPR) were enacted in consideration of the issues mentioned above [1, 2]. However, these acts made the medical data more inaccessible, and even the patients themselves could not access their own data [3]. Therefore, medical data themselves are difficult to open public and relatively small amount of data are opened to public. Furthermore, labels of medical images are difficult to obtain. Fine labels labeled by a board-certified radiologist are expensive, and weak labels labeled using previous radiologic report could be inaccurate.

Self-supervised learning (SSL) method is one kind of unsupervised pretraining method which can utilize unlabeled data. Several studies have shown that self-supervised learning can improve the performance of target tasks without using labeled data [4–7]. Similarly, there have been some approaches to overcome the expensive label issues with self-supervised learning. For example, one study could improve the target tasks by training pretext tasks training such as relative position prediction and local region reconstruction [8]. Other study improved performances in dermatology and chest radiograph (CXR) image classification tasks by adopting self-supervised pretraining [9], and another study proposed self-supervised pretraining pipeline to provide transferable initialization [10]. Furthermore, they have also shown that these approaches can overcome labels not only in the pretraining but also in the target tasks.

Some of the large datasets of CXR images has been opened to public recently [11–14]. They helped develop models by allowing many deep learning researchers to access medical images. One research group collected these data together and opened pretrained models trained on these data for transfer learning [15]. However, the size of these datasets (112–372 K) is still small compared to ImageNet, a typical deep learning computer vision benchmark of about 1.2 M size [16]. A recent study reported that they have trained self-supervised network on 100 M medical images [17]. However, various modalities of medical images are used in this study, and 1.3 M X-ray images were used in this study. Furthermore, this pretrained model or images are still not accessible to peer researchers.

Still many researchers utilize ImageNet pretrained models in medical image deep learning tasks. However, regardless of the model performances, ImageNet pretrained models in medical image might seem unreasonable to medical personnel. ImageNet models are usually pretrained on 224 × 224 resolution images, while medical images have much higher resolution. Therefore, several researches used medical image pretrained models to improve medical image deep learning tasks [10, 18–20].

For example, pulmonary nodules on medical images are defined as well lesion smaller than 30 mm [21], which can be lost in downsizing images into low-resolution such as 224 × 224. In addition, ImageNet images are 3-channel RGB images, while radiologic images are usually 1-channel grayscale images. Therefore, ImageNet pretrained models can be less reliable in medical image due to the discrepancy in the settings between pretraining and target tasks. Furthermore, researchers might need more computational resources, such as GPU memories, since they typically resize 1-channel medical images to 3-channel images when using ImageNet pretrained models.

In this study, we propose chest X-ray pre-trained model via self-supervised contrastive learning (CheSS), which has been pretrained using considerable amount of CXR images and is freely accessible to researchers.

## Materials and Methods

This retrospective study was conducted according to the principles of the Declaration of Helsinki and according to current scientific guidelines. The Institutional Review Board Committee approved the study protocol. The Institutional Review Board Committee waived the requirement for informed patient consent due to the retrospective nature of this study.

### Dataset Preparation and Image Pre-processing

#### Dataset

For training an upstream method, 4.8 M CXR images were obtained retrospectively from a tertiary hospital in South Korea. A total of 3.6 M adult CXR images were collected from 2011 to 2018. Next, 1.2 M pediatric CXR images were collected from 1997 to 2018.

In downstream tasks, CXR images with 6-class diseases which were confirmed by near computed tomography (CT) scans within 1 month were first collected from the same hospital but independently of the upstream method for the multi-class classification. CXR images of 2571 healthy subjects and 3417 patients were obtained, with the latter including 944, 1540, 280, 1364, and 330 patients with “nodule,” “consolidation,” “interstitial opacity,” “pleural effusion,” and “pneumothorax,” respectively. Chest CT images were used to confirm the presence of normal and abnormal nodules (including masses), or interstitial opacities in the dataset, as well as pleural effusion and pneumothorax, were determined by the consensus of two thoracic radiologists using CXR images and corresponding chest CT images [22].

Second, we used the CheXpert [12] dataset, which contains CXR images for the multi-label classification. Like the original CheXpert leaderboard [23, 24], “atelectasis,” “cardiomegaly,” “consolidation,” “edema,” and “pleural effusion” diseases were selected for validation test. Third, we collected 4033 adult posterior-anterior pairs of rib-preserved and rib-suppressed bone suppression images, generated using the Bone Suppression™ software (Samsung Electronics Co., Ltd.) [25] for the bone suppression. Finally, we used images of patients with “nodule” from the 6-class dataset for the nodule generation.

#### Image Preprocessing

All CXR images were resized into 512 × 512 pixels. Next, to alleviate the high intensity of L/R markers in CXR images, we limited the CXR images’ maximum pixel value to the top 1%-pixel value of each CXR image [26].

### Training Visual Representation of CXR as an Upstream Method

**Fig. 1:**
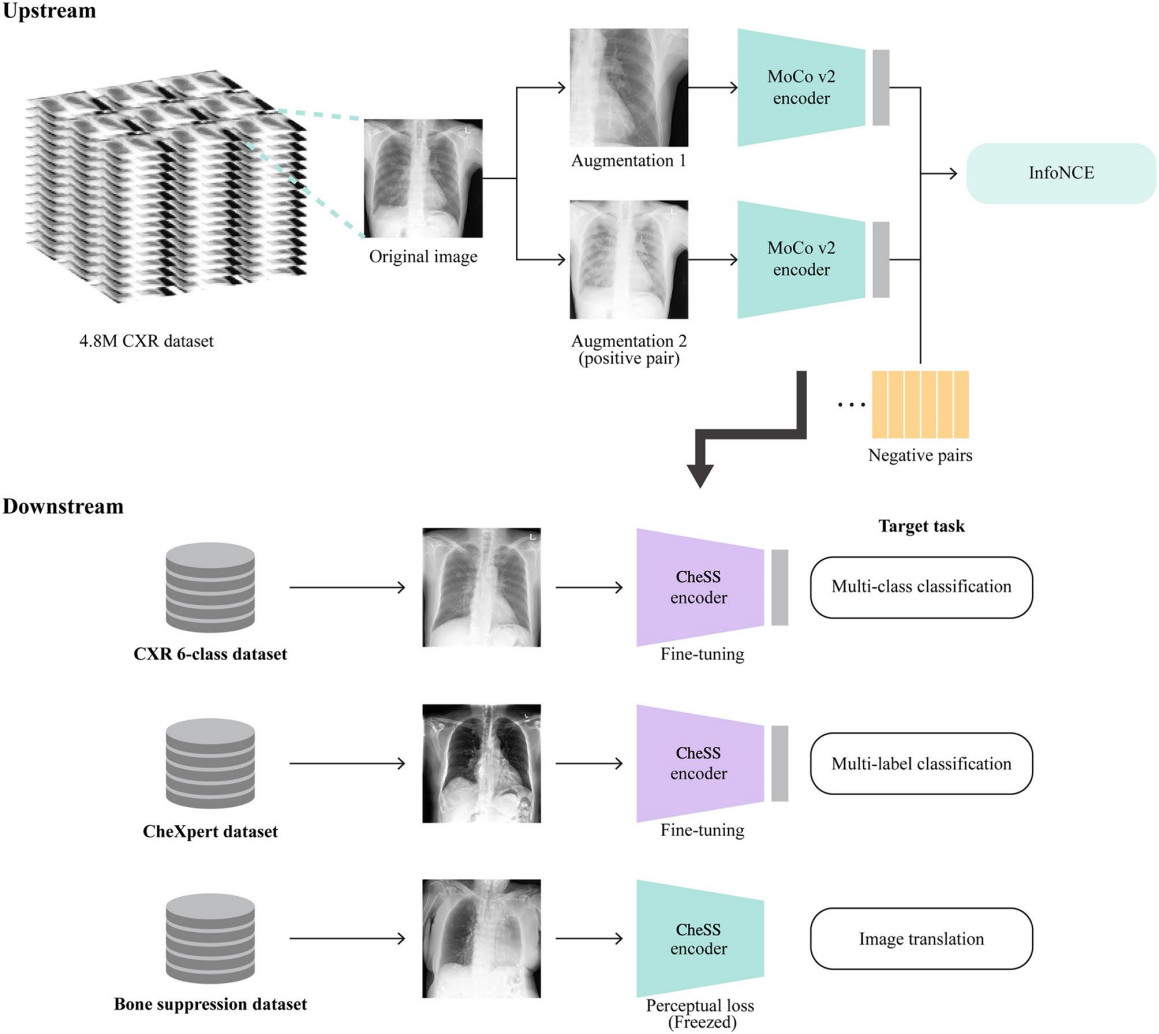
Overall workflow of our method consisting of upstream and downstream methods. In the upstream method, a model in MoCo v2 manner was trained. In downstream tasks, the transfer learning with pretrained weights of upstream model was used to train multi-class classification, multi-label classification, and image-to-image translation

We trained the self-supervised contrastive pretraining method with unlabeled images using MoCo v2 [6] to learn visual representations of CXR. The upstream method maximizes the similarity between two views of the same CXR images (positive pair) and minimizes the similarity between different CXR images (negative pairs). Our method is illustrated in Fig. 1.

For upstream training, 8 GPUs (Tesla V100) and a batch size of 256 were used. All models were implemented using PyTorch framework. In this study, a 50-layer residual network (ResNet) [27], one of the most commonly used networks in deep learning, was used. The SGD optimizer with a learning rate of 1e − 5, momentum of 0.9, and weight decay of 1e − 4 was adopted. Shifting, zooming, rotation, blur, sharpening, Gaussian noise, cutout, and optical distortion were used for data augmentation. To train the model, we used InfoNCE [6, 28] as an unsupervised objective function to train the encoder networks that represent queries and keys. The loss function is calculated as follow:

$${\mathcal{L}}_{\mathcal{q}}=-\mathrm{log}\frac{\mathrm{exp}\left(q\cdot {k}_{+}/\uptau \right)}{{\sum }_{i=0}^{K}\mathrm{exp}\left(q\cdot {\mathrm{k}}_{\mathrm{i}}/\uptau \right)}$$

where $q,{k}_{+}$, and ${k}_{i}$ represent a query, a positive key that matches the query, and all keys including both positive and negative keys, respectively. In addition, we adopted MoCo v2 [6], which performs momentum updates by storing a dictionary queue structure of data samples that can efficiently use the high resolution’s CXR information. Finally, training our model took about 8 weeks.

### Evaluation via Various Downstream Target Tasks

To evaluate our pretrained model, many downstream tasks were conducted as follows. First, to compare the effectiveness of our pretrained weight with ResNet50, which has been trained in a supervised manner using ImageNet-1 k dataset or randomly initialized weight, we conducted fine-tuning on the CXR 6-class dataset. To simulate various clinical situations, we applied various data imbalanced settings in composing the training dataset. The details on the amount of data and the settings are summarized in Table 1.

**Table 1:** Dataset settings used in CXR 6-class classification. The same number of images for each class was sampled for the undersampled dataset. Normal, nodule, and consolidation images were additionally sampled for the modified dataset, while the interstitial opacity images were simply duplicated because there was no additional data for interstitial opacity

	Normal	Nodule	Consolidation	Interstitial opacity	Pleural effusion	Pneumothorax
Full	2,515	888	1,484	224	1,308	274
Undersampled	224	224	224	224	224	224
Modified	336	560	560	448	224	224
Validation	28	28	28	28	28	28
Test	28	28	28	28	28	28

Second, the CheXpert dataset [12] was used to evaluate the generalizability of our method. This task was also compared among three models with the randomly initialized weight, ImageNet pretrained weight, and our pretrained weight. Furthermore, stress tests using data fractions of 1%, 10%, and 50% were also conducted to demonstrate that data shortage can be supplemented using our pretrained weight.

Finally, since the perceptual loss from task-specific feature extractor has been used recently [29–31], image-to-image translation tasks were conducted to suggest potential usage of our pretrained model for perceptual loss [32]. Bone suppression and nodule generation were conducted to demonstrate that our pretrained model can be used for perceptual loss. Details of the downstream training strategy can be found in the Supplementary materials.

## Results

### CXR 6-Class Classification

Various data settings were assumed to consider the actual data distribution in the real clinical environment. Severe data imbalance was established in the initial setting, with maximum 1540 and minimum 280 images. The validation and test datasets were made common for all experiments, for a fair comparison.

Table 2 shows the result of all experiments conducted. CheSS showed statistically significant better compared to those of the ImageNet pretrained model (*P*-value < 0.001) and randomly initialized model (*P*-value < 0.001) in Stuart-Maxwell test.

**Table 2:** Accuracies of 6-class classification model with multiple data imbalance simulations

	Full	Undersampled	Modified
Scratch	0.375***	0.464***	0.369***
ImageNet	0.554**	0.405**	0.398***
Ours (CheSS)	**0.631**	**0.554**	**0.654**

Stuart-Maxwell test was conducted to compare scratch (randomly initialized), ImageNet, and our (CheSS) pretrained models. The bold text indicates the best performance

*p < 0.05; **p < 0.01; ***p < 0.001

A full dataset was set up to compare the capabilities to overcome the data imbalance of each pretrained weight. An undersampled dataset was set up to compare the model performances in the fair but scarce amount of data. Finally, the modified dataset was set, in which the amount of data was set according to the difficulties of each class in the dataset. Because ImageNet can sometimes have worse performance than scratch depending on image size and dataset size [33], it is not surprising that ImageNet can perform slightly worse in some settings.

### CheXpert Multi-Label Classification

Stress tests of multiple data fractions were conducted considering the data shortage in an actual research environment. Data fractions of 1%, 10%, 50%, and 100% were established to compare each pretrained weight’s capabilities for evaluating overcoming performances in data shortage. For the stability and the reproducibility of the data stress test results, fine-tuning experiments on small data fractions were repeated multiple times with different random samples and averaged. Common unseen test datasets in all experiments were fixed for a fair comparison.

**Fig. 2:**
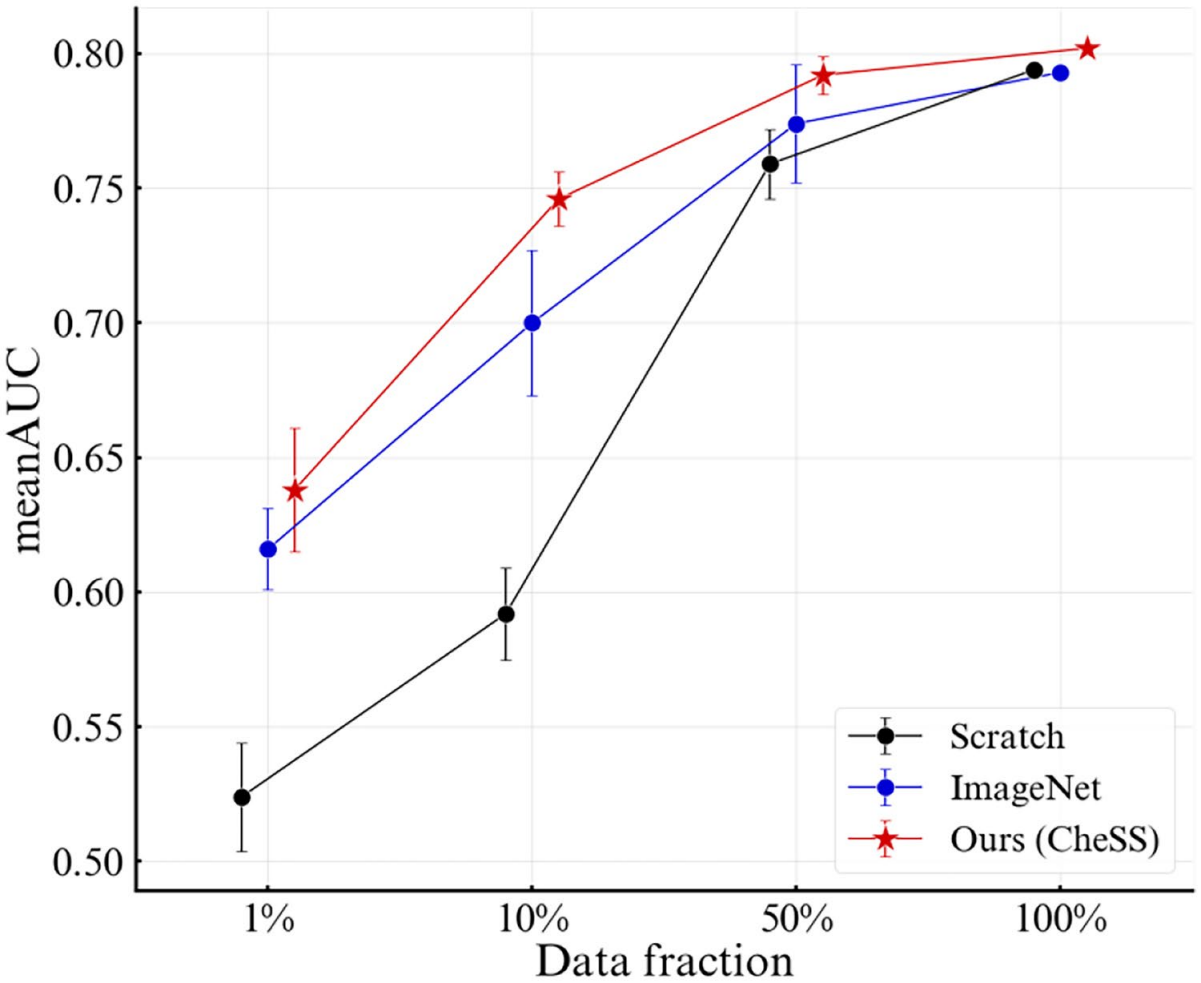
Mean area under receiver operating characteristics curve (AUCs) and standard deviations (SDs) on multiple data fraction fine-tuning with the weights of scratch, ImageNet, and CheSS pretrained models. Data fractions of 1%, 10%, and 50% were experimented on 10 times with different random samples. The result of the full dataset is presented only with AUC

Figure 2 depicts the results of the full dataset experiment and data stress tests of multiple data fractions. In the full dataset, CheSS showed the best mean area under receiver operating characteristics curve (AUC) of 0.808, while the ImageNet pretrained model showed 0.795, and the scratch model showed 0.794. The detailed results of the full dataset experiment are summarized in Supplementary Table 1. Furthermore, in the 1% data fraction test, CheSS, ImageNet pretrained model, and scratch achieved a mean AUC of 0.638 ± 0.023, 0.616 ± 0.015, and 0.524 ± 0.020, respectively. Paired *t*-tests were conducted to compare the results. Quantitative results are summarized in Table 3.

**Table 3:** Mean AUCs and SDs on 1%, 10%, and 50% data fraction that were experimented on 10 times with the weights of CheSS, ImageNet, and scratch. The result of the full dataset was presented only with AUC

	1%	10%	50%	100%
Scratch	0.524 ± 0.020***	0.592 ± 0.017***	0.759 ± 0.013***	0.794
ImageNet	0.616 ± 0.015*	0.700 ± 0.027**	0.774 ± 0.022*	0.795
Ours (CheSS)	**0.638 ± 0.023**	**0.746 ± 0.010**	**0.790 ± 0.012**	**0.807**

Mean AUCs were compared using paired *t*-tests. The bold text indicates the best performance

*p < 0.05; **p < 0.005; ***p < 0.001

### Qualitative Results on Classification Results

**Fig. 3:**
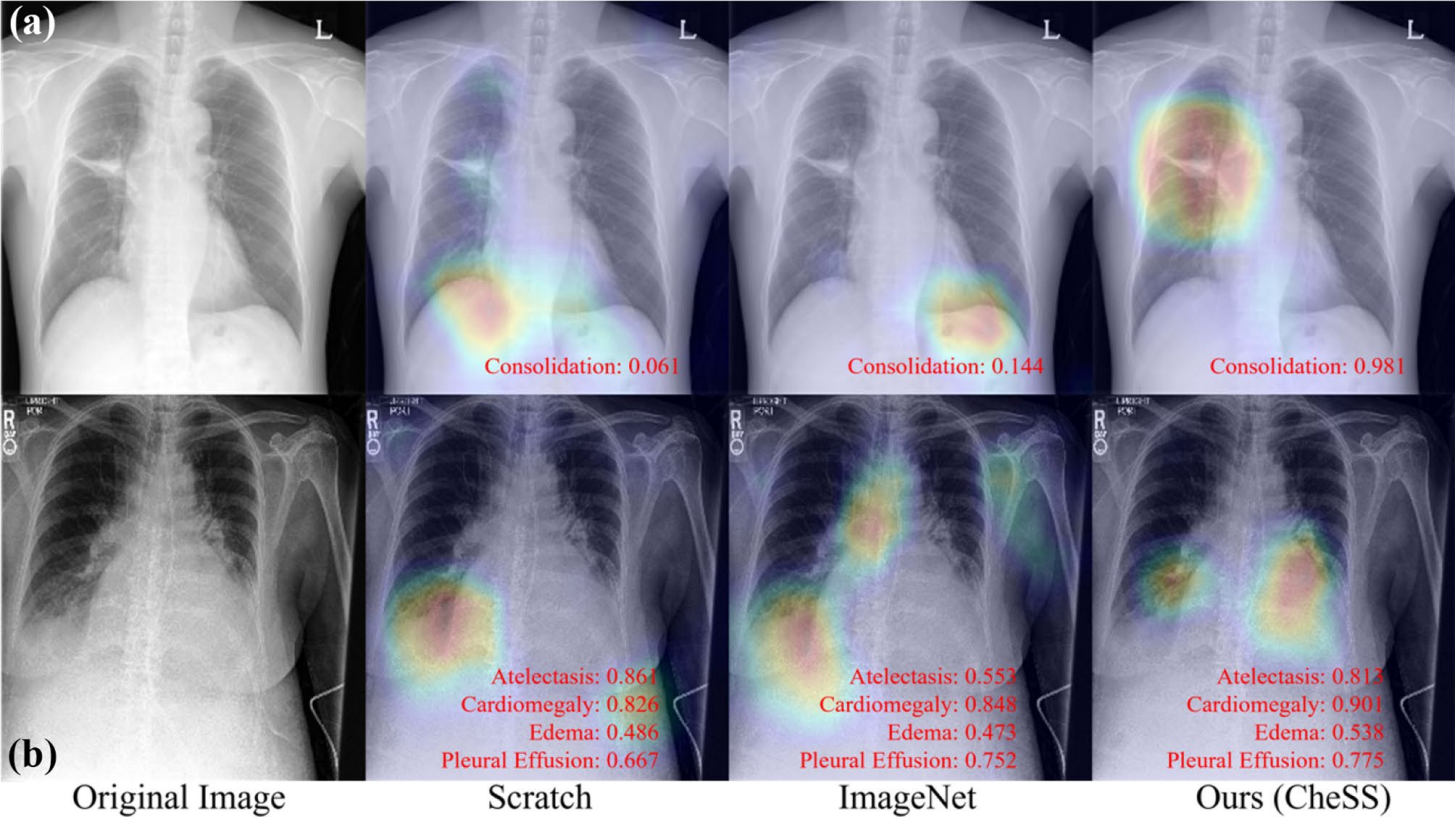
Grad-CAM acquired from **a** 3.1 6-class classification in CXR and **b** 3.2 CheXpert multi-label classification. Ground truth label for **a** is consolidation, and labels for **b** are atelectasis, cardiomegaly, edema, and pleural effusion

Saliency maps acquired using gradient-weighted class activation map (Grad-CAM) [34] were used to compare the qualitative results. Figure 3 depicts the results of Grad-CAM of each model. The red text in Fig. 3 is the logit value for each model (scratch, ImageNet, CheSS) of (a) 6-class classification and (b) multi-label classification, respectively. In Fig. 3a, the logit value for the consolidation label in the image was the highest in our model at 0.981. Also, in Fig. 3b, the logit values of our model were high at 0.901, 0.538, and 0.775 for the cardiomegaly, edema, and pleural effusion labels.

### Image-to-Image Translation using Perceptual Loss

Bone suppression and nodule generation tasks were conducted to evaluate the potential usage of CheSS for perceptual loss. Dilated U-Net [35, 36] was used for bone suppression. Structural similarity index measure (SSIM) [37], peak signal to noise ratio (PSNR), and root-mean-square error (RMSE) were used for the quantitative evaluation. Moreover, dilated U-net without perceptual loss was additionally compared. SPADE [38, 39] with perceptual loss was used in the nodule generation task. Fréchet inception distance (FID) [40] was used for the quantitative results. CheSS pretrained and ImageNet pretrained ResNet [27] encoders for perceptual loss were mainly compared in this section.

Table 4 shows the quantitative results of two-generation downstream tasks with perceptual loss. In bone suppression, CheSS showed statistically significant results in terms of PSNR, SSIM, and RMSE when compared with the ImageNet pretrained model and no perceptual loss. The perceptual loss of CheSS also showed better results in terms of FID in nodule generation compared with the ImageNet pretrained model.

**Table 4:** Quantitative results of image-to-image translation

	Bone suppression^a^			Nodule generation
	PSNR↑	SSIM↓	RMSE↓	FID↓
No perceptual	32.01***	0.946***	5.544***	-
ImageNet	34.99***	0.976***	4.410***	24.06
Ours (CheSS)	**37.77**	**0.977**	**3.301**	**17.07**

*SSIM* structural similarity index measure, *PSNR* peak signal to noise ratio, *RMSE* root-mean-square error, *FID* Fréchet inception distance. The bold text indicates the best performance

*p < 0.05; **p < 0.005; ***p < 0.001

^a^Paired *t*-test was conducted to compare perceptual loss of each model

**Fig. 4:**
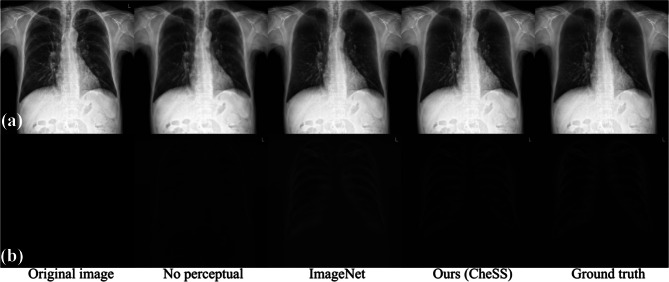
Example bone suppression images with perceptual loss, without perceptual loss of ImageNet pretrained encoder, and with perceptual loss of CheSS pretrained encoder. Bone suppression images are shown in **a**, and the residual maps (subtraction between the original image and the bone suppression image) are shown in **b**

The qualitative results for bone suppression are shown in Fig. 4, and the results for nodule generation are shown in Supplementary Fig. 1.

## Discussion

We trained CheSS using a SSL method on a large-scale dataset of 4.8 M CXR images. In this study, we evaluated CheSS with many downstream tasks. CheSS showed better performance than scratch and the ImageNet pretrained model in many downstream tasks. CheSS showed decent transferability in multiple datasets and data settings in multi-class and multi-label classification. Data imbalance and data shortage can be supplemented with our CheSS pretrained weight. Furthermore, CheSS does not need a strict preprocessing principle as mentioned in “Image preprocessing” section. The same preprocessing in the upstream method might be optimal for using CheSS. Still, it showed good transferability on CheXpert, which has a different preprocessing principle from our method, as shown in Supplementary Fig. 2. The potential usage of an CheSS pretrained encoder for perceptual loss was also demonstrated in this study. We have shown that multiple data issues, such as data imbalance and data shortage, can be supplemented with our open pretrained weight.

Many researchers utilize ImageNet pretrained models in medical image deep learning tasks. However, regardless of the model performances, ImageNet pretrained models might seem unreasonable to medical personnel. The first reason for that is ImageNet models are usually pretrained on 224 × 224 resolution images, while medical images have much higher resolution. Second, pulmonary nodules on medical images are defined as lesions smaller than 30 mm [21], which can be lost while downsizing images to low resolutions such as 224 × 224. Third, ImageNet images are 3-channel (RGB) images, while radiologic images are usually 1-channel (grayscale) images. Thus, ImageNet pretrained models can be less reliable for medical images owing to the large discrepancy between pretraining and target tasks. In addition, researchers might need more computational resources, such as GPU memories, since they typically resize 1-channel (grayscale) medical images to 3-channel (RGB) images when using ImageNet pretrained models.

Our study has several limitations. First, external validation in the classification method was performed with only one dataset owing to limited time and resources. A further study is required to confirm the universal transferability of CheSS. Second, we did not use dense prediction methods such as object detection and semantic image segmentation. However, the qualitative results show acceptable localizing performances. A further study of dense prediction is also needed to verify our method’s capabilities of localizing a region of interest. Third, more ablation studies, stress tests, and parameter searching are needed to evaluate the performance of CheSS weights. Finally, several studies [6, 7, 28] have shown that using a batch size of more than 1000 in the upstream task leads to good performance. However, the size of the images used in these papers was set to 224 × 224, while ours was 512 × 512 in consideration of the characteristic of medical imaging with high resolution [21]. Due to limitations on resources and time, we were unable to experiment with various batch sizes. In the further studies, we will include the ablations study of various batch sizes on a self-supervised network for high-resolution medical image analysis.

## Conclusion

This study showed the decent transferability of CheSS weights. This open model can help researchers overcome data imbalance, data shortage, and inaccessibility of medical image datasets. CheSS can also be used for perceptual loss in image-to-image translation.

## Supplementary Information

Below is the link to the electronic supplementary material.

Supplementary file1 (DOCX 7246 KB)
