# Supplementary material for: CheSS: Chest X-Ray Pre-trained Model via Self-supervised Contrastive Learning
Source: J Digit Imaging. 2023 Jan 26;36(3):902–10. doi: 10.1007/s10278-023-00782-4 (PMC10287612; doi:10.1007/s10278-023-00782-4)
Supplement: Supplementary file 1 — Supplementary file1 (DOCX 7246 KB) [file 10278_2023_782_MOESM1_ESM.docx]

**CheSS: Chest X-ray pre-trained model via Self-Supervised contrastive learning**

**Downstream training strategy**

**CXR 6-class dataset**

The same architecture as upstream was used, and the model was initialized by applying fine-tuning to the weights that were trained by upstream. For training, we used the SGD optimizer with a learning rate of 5e-2 and weight decay of 1e-5 to train the model in all experiments. One GPU (NVIDIA Titan RTX 24GB) and a batch size of 48 were used. And the augmentation strategy was the same as that of the upstream task.

**CheXpert dataset**

The same architecture as upstream was used, and the model was initialized by applying fine-tuning to the weights that were trained by upstream. we used the SGD optimizer with a learning rate of 1e-3 and weight decay of 1e-5 to train the model in all experiments. Two GPU (NVIDIA Titan RTX 24GB) and a batch size of 100 were used. And the augmentation strategy was the same as that of the upstream task.

**Bone suppression and nodule generation dataset**

We used the weights trained by upstream as the feature extractor and matched each of the features with the output of the final layer of the five convolution blocks. For training, we used the Adam optimizer with a learning rate of 2e-3 and weight decay of 1e-5 to train the model in all experiments. One GPU (NVIDIA RTX A6000) and a batch size of 16 were used in the bone suppression and two GPU (NVIDIA RTX A6000) and a batch size of 8 were used in the nodule generation.

Supplementary Table 1. Detailed results of fine-tuning on full dataset with CheSS pre-trained model weight, ImageNet pre-trained model weight, and scratch model.

|  | Atelectasis | Cardiomegaly | Consolidation | Edema | Pleural effusion |
| --- | --- | --- | --- | --- | --- |
| Scratch | 0.785 | 0.843 | 0.831 | 0.698 | 0.815 |
| ImageNet | 0.736 | 0.917 | 0.734 | 0.716 | 0.870 |
| Ours (CheSS) | 0.773 | 0.903 | 0.781 | 0.697 | 0.881 |


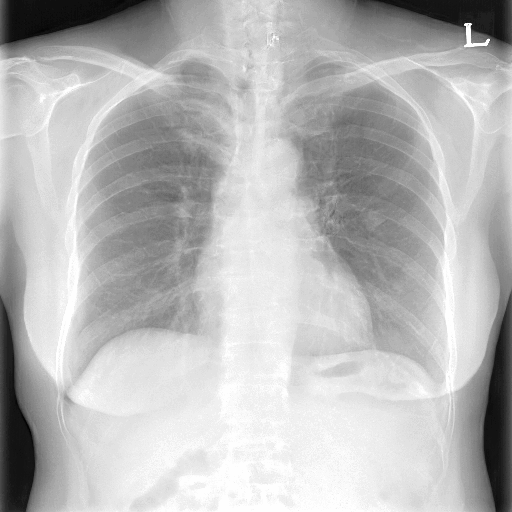

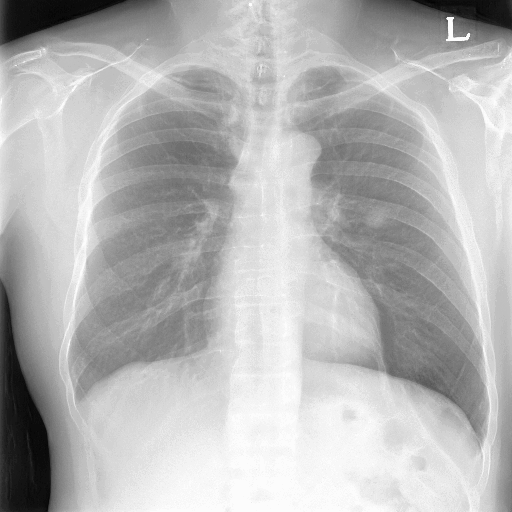


Supplementary Fig. 1-a. Example animated images of nodule generation using perceptual loss with ImageNet pretrained encoder (left) and perceptual loss with CheSS pretrained encoder (right). When using perceptual loss with ImageNet pretrained encoder (left), the mediastinal area remains fixed, which indicates low diversity. In addition, the scapular area in some images is not generated well, which indicates low fidelity. Nodule generation images using perceptual loss with CheSS pretrained encoder (right) show better images in terms of both diversity and fidelity.


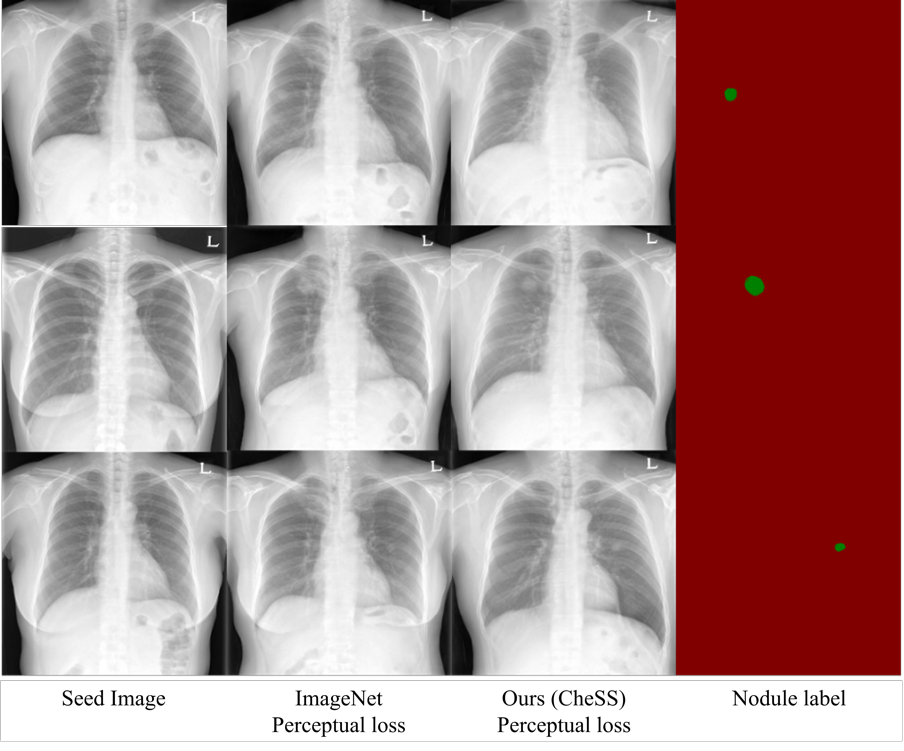


Supplementary Fig. 1-b. Example nodule generation images using perceptual loss with ImageNet pretrained encoder and perceptual loss with CheSS pretrained encoder.


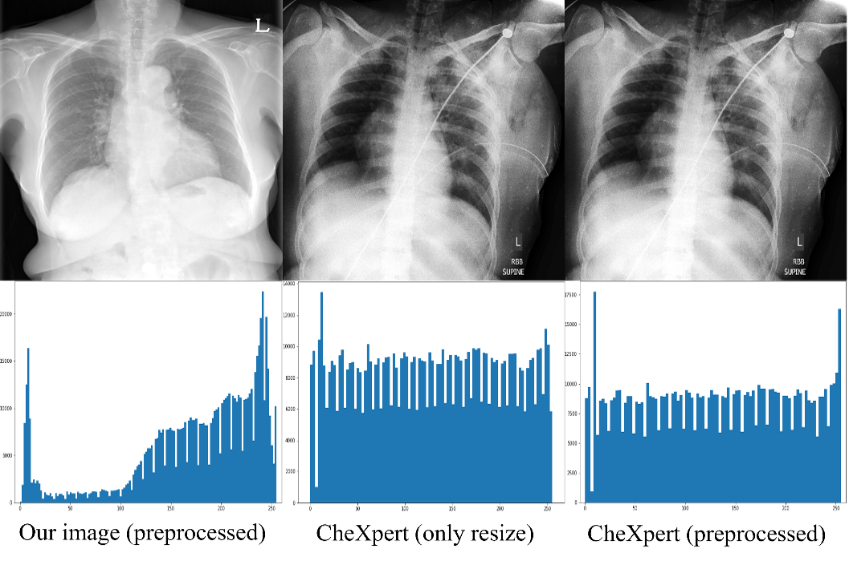


Supplementary Fig. 2. Example image and histogram of the image used in self-supervised learning and CheXpert image. Note that histogram of our image shows the typical multimodal peak of CXR, and CheXpert image shows the equalized histogram.
